# Gut Serpinome: Emerging Evidence in IBD

**DOI:** 10.3390/ijms22116088

**Published:** 2021-06-04

**Authors:** Héla Mkaouar, Vincent Mariaule, Soufien Rhimi, Juan Hernandez, Aicha Kriaa, Amin Jablaoui, Nizar Akermi, Emmanuelle Maguin, Adam Lesner, Brice Korkmaz, Moez Rhimi

**Affiliations:** 1 Microbiota Interaction with Human and Animal Team (MIHA), Micalis Institute, AgroParisTech, Université Paris-Saclay, INRAE, 78350 Jouy-en-Josas, France; hela.mkaouar@inrae.fr (H.M.); vincent.mariaule@inrae.fr (V.M.); soufienrhimi@yahoo.fr (S.R.); aicha.kriaa@inrae.fr (A.K.); amin.jablaoui@inrae.fr (A.J.); akerminizar1990@gmail.com (N.A.); emmanuelle.maguin@inrae.fr (E.M.); 2 Department of Clinical Sciences, Nantes-Atlantic College of Veterinary Medicine and Food Sciences (Oniris), University of Nantes, 101 Route de Gachet, 44300 Nantes, France; juan.hernandez@oniris-nantes.fr; 3 Faculty of Chemistry, University of Gdansk, Uniwersytet Gdanski, Chemistry, Wita Stwosza 63, PL80-308 Gdansk, Poland; adam.lesner@ug.edu.pl; 4 INSERM UMR-1100, “Research Center for Respiratory Diseases” and University of Tours, 37032 Tours, France; brice.korkmaz@inserm.fr

**Keywords:** inflammatory bowel diseases, gut serpinome, proteases, gut microbiota, holobiont

## Abstract

Inflammatory bowel diseases (IBD) are incurable disorders whose prevalence and global socioeconomic impact are increasing. While the role of host genetics and immunity is well documented, that of gut microbiota dysbiosis is increasingly being studied. However, the molecular basis of the dialogue between the gut microbiota and the host remains poorly understood. Increased activity of serine proteases is demonstrated in IBD patients and may contribute to the onset and the maintenance of the disease. The intestinal proteolytic balance is the result of an equilibrium between the proteases and their corresponding inhibitors. Interestingly, the serine protease inhibitors (serpins) encoded by the host are well reported; in contrast, those from the gut microbiota remain poorly studied. In this review, we provide a concise analysis of the roles of serine protease in IBD physiopathology and we focus on the serpins from the gut microbiota (gut serpinome) and their relevance as a promising therapeutic approach.

## 1. Introduction

Inflammatory bowel diseases (IBDs) comprise the chronic relapsing inflammatory disorders Crohn’s disease (CD) and ulcerative colitis (UC). Both are thought to arise in genetically prone individuals under the influence of environmental factors that trigger excessive activation of the host immune response. The gut microbiota is currently considered an important regulator of innate and adaptive immunity [1]. Its composition and functions are modulated by many environmental factors, such as diet, xenobiotics and infectious and toxic agents [2,3]. The changes in the microbiota induced by the environmental perturbations of the recent decades could contribute significantly to the global epidemic evolution of IBD. There is increasing evidence for the involvement of imbalanced host–gut microbiota interactions in IBD pathogenesis [4]. It has been shown that dysbiosis induces multiple deleterious processes, including an alteration of the fermentation-derived products such as carbohydrates, vitamins and short-chain fatty acids [5]. Dysregulation of bile acid biotransformation is also reported [6]. 

The imbalance of proteolytic activity in the digestive tract has been scarcely investigated till now, but it constitutes an important modality of dialogue between the microbiota and the host. Recent studies have involved proteases from the host and the gut microbiota in the pathogenesis of IBD. Indeed, both intestinal and fecal samples from CD and UC patients showed excessive serine protease activity as compared to healthy controls [7]. This uncontrolled activity is likely to play key roles in a variety of signaling pathways, inflict tissue damage and exacerbate gut inflammation [8,9,10]. The regulation of such proteases by their endogenous inhibitors would therefore represent a promising therapeutic alternative for treating or controlling IBD. 

Serine protease inhibitors (serpins) comprise the most widely distributed superfamily of protease inhibitors, and their anti-inflammatory properties have been demonstrated in inflammatory disorders [11,12,13]. Over the past few decades, the attention of the medical and research community has been focused on host serpins for disease management. Meanwhile, the role of their gut bacterial counterparts has been dismissed. A broader view should include gut microbial serpins with the potential to act as suicide substrates targeting host and microbial proteases involved in IBD. This would cover the full pan-microbial genome that reflects the total number of non-redundant serpin-encoding genes in the human gut microbiota. 

In this review, we present an overview of the role of the gut microbiota and proteases in IBD; we highlight the importance of serpins, mainly those produced by the gut microbiota, and propose to group them under the term gut serpinome.

## 2. Impact of Inflammatory Bowel Diseases and Available Treatments

The prevalence of IBD continues to evolve around the world. Until the 2010s, the incidence of IBD in industrialized countries (Europe, North America, Oceania) continued to grow, reaching an average of 10 new cases per 100,000 inhabitants per year [14]. There is a significant disparity in the distribution of new cases, with a north–south and west–east gradient in Europe [15]. Over the same period, the disease affected increasingly younger patients, with a significant increase in incidence in children and adolescents [16]. Since 1990, newly industrialized countries in Africa, Asia and South America have shown a subtle increase in IBD incidence with, also, a heterogeneous distribution by region [14,17,18]. Considering the chronicity of CD and UC and the relatively low mortality, the prevalence of the disease continues to increase, reaching more than 6.8 million patients in Europe and the US today [19]. 

Different types of immunosuppressant drugs are used alone or in combination to induce and maintain remission [20]. The current therapeutic strategy consists of a gradual increase in treatment intensity, from medication to surgery, depending on the patient’s response. Medical treatment includes aminosalicylates, immunomodulators, steroids, biological treatments (anti-TNFα and anti-α4β7) and leukapheresis. Half of the patients with CD or UC will have to undergo surgery (colectomy, anastomosis, deviation) during their lifetime [21]. In addition to the cumbersome treatment and the potential for side effects, none of them are curative. 

Several studies showed that IBDs strongly affects quality of life. In fact, they induce psychological distress related to the symptoms and to the uncertainty of the course of the disease. In addition, fatigue, which is a common feature of the disease, impacts daily activities as well as leisure, professional, family and private life [22,23]. 

Besides human consequences for the patient and their relatives, IBDs have a significant economic impact. A recent study carried out by the Crohn’s & Colitis Foundation in 2020 showed that annual mean health care costs were three-fold higher in IBD patients than those without IBD (around USD 23,000 vs. USD 6956/patient/year) [24]. Patients with IBD also incurred more than twice the out-of-pocket expenditures and had lifelong indirect costs related to disease management [24].

All these data stress the increasing cost burden of IBD patients and emphasize the need for novel cost-effective therapeutic strategies. 

## 3. Role of the Gut Microbiota in the Pathogenesis of IBD

**Figure 1:**
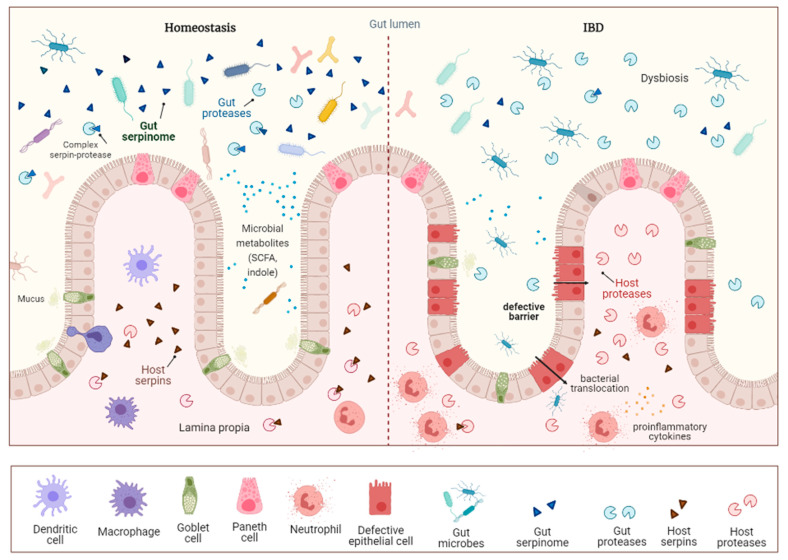
Schematic illustration of gut serpinome involvement in intestinal homeostasis and IBD. During homeostasis, the gut microbiota elicits an immune tolerance phenotype in the host. The activity of luminal serine and cysteine proteases is tightly regulated by their specific serpins of both gut microbial and host origin. A key feature of IBD is the alteration of the composition of the gut microbiota, dysbiosis, characterized by the decrease in microbial diversity with a loss of beneficial symbionts and the expansion of pathobionts. The dysregulation of the proteolytic balance with an increased protease activity over serpins alters the intestinal barrier and exacerbates inflammation. SCFA: short-chain fatty acid.

Trillions of microorganisms reflecting all kingdoms of life inhabit the gastrointestinal tract (GIT) [25,26,27]. This gut microbiota represents a complex community whose members interact with each other and with the host to control several processes essential for maintaining host homeostasis and health [28,29,30]. Alterations in the composition and function of the gut microbiota have been reported in many studies related to digestive inflammation [31,32,33,34,35,36]. This intestinal dysbiosis was shown to influence the production of microbiota-derived metabolites and further impact the microbiota–host crosstalk (Figure 1). 

Dysbiosis may involve the reduction of the overall microbial richness, diversity and/or a loss of beneficial microorganisms. However, since a common understanding of what constitutes a healthy microbiota is still lacking, it is unclear how to delineate a dysbiotic one. Previous reports have linked reduced microbial diversity to the disease, referring to the loss of metabolic redundancy [32,37]. The loss of *Faecalibacterium prausnitzii*, for instance, which belongs to Clostridium cluster IV, has been typically observed in patients with CD and UC [38,39,40]. *F. prausnitzii* is known for its anti-inflammatory properties related to NF-kB inhibition, stimulation of anti-inflammatory cytokine secretion such as IL-10 [41,42] and production of the short-chain fatty acid (SCFA), butyrate [43]. SCFAs are the end-products of microbial fermentation, which regulate the immune response and contribute to intestinal integrity. Consistent with the depletion of SCFA-producing bacteria (*F. prausnitzii* and specific *Roseburia* species) in IBD, a reduction in fecal SCFA levels was noted in a metabolomic analysis of IBD patients [44,45]. Lower levels of tryptophan and its metabolites were detected as well in patients with IBD as compared to healthy controls [46]. Evidence suggests that tryptophan metabolites of microbial origin could exert anti-inflammatory effects [46] and regulate the homeostasis of the gut microbiota. 

Dysbiosis may also be linked to the expansion of potentially harmful microbes or pathobionts. Multiple studies revealed a higher prevalence of *Bacteroides fragilis* in IBD samples [47,48]. Strains of enterotoxigenic *B. fragilis* secrete various virulence factors that target the epithelial barrier and thereby contribute to intestinal inflammation [49,50]. Adherent Invasive *Escherichia coli* (AIEC) [51,52], *Mycobacterium avium* subsp. *paratuberculosis* [53], *Enterococcus faecalis* [54,55], *Salmonella typhimurium* [56] and many other bacterial pathogens have also often been associated with IBD.

Understanding the drivers of disease severity and the impact of bacteria and/or their metabolites on IBD progression will uncover cardinal targets and novel therapeutic approaches.

## 4. Proteases as Key Targets in Intestinal Inflammation

Proteases have received increasing attention over the last few years. The importance of these enzymes as potential therapeutic targets or biomarkers for IBD has led to extensive efforts in the screening of their specific inhibitors. Here, we examine the impact of host and gut microbial serine and cysteine proteases which are controlled by microbial serpins.

### 4.1. Role of Human Proteases in IBD

Serine proteases are involved in a multitude of biological processes, such as the immune response, digestion and blood coagulation, and are key signaling molecules in gastrointestinal physiology and in the inflammatory response [57]. Host cellular sources of serine proteases encompass a wide range of cell types, from intestinal epithelial cells to resident and infiltrated cells. Immune cells such as neutrophils and mast cells are key sources of serine proteases, which are stored in their granules. In fact, tryptase, chymase, cathepsin G (CatG) and granzyme B are secreted by mast cells [58], while neutrophils release neutrophil elastase (NE), proteinase 3 (PR3) and CatG at the site of inflammation [59]. Under physiological conditions, serine proteases’ activity is tightly regulated, while a disequilibrium in their proteolytic activity is linked to several gastrointestinal disorders, including IBD. Indeed, an increase in serine protease expression and activity has been demonstrated in the colonic tissue and fecal samples of IBD patients compared to healthy controls [7,10]. Such deregulated proteolytic activity was shown to participate in the inflammatory response and to cause structural and functional alterations in the gut epithelium through (i) the activation of protease-activated receptor (PAR), (ii) the cleavage of junctional protein and (iii) the processing of cytokines and chemokines. A recent study demonstrated that the pro-inflammatory effects of colonic thrombin, whose activity showed a 100-fold increase in the biopsies of IBD patients [60], are mediated through the activation of PAR1 and PAR4 [61]. Only PAR1 inhibition prevents 2,4,6-Trinitrobenzenesulfonic acid (TNBS)-induced colitis in rats. Among mast cell serine proteases, tryptase was shown to activate PAR2 and the subsequent Akt/mTOR pathway, therefore promoting IBD-induced intestinal fibrosis [62]. PAR4 activation by CatG triggers altered epithelial permeability and inflammation via myosin light-chain kinase (MLCK) activation, leading to myosin light chain (MLC) phosphorylation and tight junction (TJ) destabilization [63]. Junctional proteins, along with PAR, are key elements in the inflammatory response and in increased permeability mediated by serine proteases. For instance, chymase causes enhanced epithelial permeability through the redistribution of the TJ proteins ZO-1 and occludin [64]. Leukocyte transmigration to the inflammation site is associated with the direct proteolytic degradation of the vascular endothelial cadherin by CatG and NE [65]. Furthermore, the processing of CXCL-5 and CXCL-8 chemokines by CatG and PR3 results in higher chemotactic proprieties towards neutrophils [66].

Cysteine proteases, widely distributed among living organisms, possess a catalytic Cys–His–Asn triad, where the cysteine is responsible for nucleophilic attack. Cysteine proteases are involved in numerous biological processes, such as senescence, apoptosis, inflammation, major histocompatibility complex class II (MHC II) immune responses and extracellular matrix remodeling [67]. Among the cysteine proteases, caspases and cathepsins have been studied for their potential role in IBD pathogenesis. In humans, the caspase family is composed of 12 members and most of them are key actors in programmed cell death, proliferation and inflammation [68]. At gene level, the caspase 9 gene has been suggested to be an IBD susceptibility gene [69]. In a recent study, altered expression of inflammatory caspases (caspase 1, 4 and 5) has been shown to be involved in intestinal inflammation in IBD patients [70]. The human genome encodes for 11 cysteine cathepsins, which have been shown to have a role in chronic inflammatory diseases [71]. At the very least, cathepsin B and L expression is increased in intestinal macrophages in the inflamed mucosa of IBD patients and their combined inhibition resulted in the reduction of experimental colitis severity [72]. 

With these data, serine and cysteine protease inhibition might represent a promising alternative to treat IBD. Confirmation of this assumption requires an investigation of the role of their microbial counterparts in inflammation.

### 4.2. Role of Microbial Proteases in IBD

Proteases have been widely explored in several pathological conditions. However, only recently, the gut proteases have emerged as a functional partner playing key roles in health and disease. These enzymes are essential to bacterial viability, the stress response as well as pathogenicity [73]. Many proteases, including high-temperature serine protease A (HtrA), are tightly regulated to prevent intrusive bacterial growth and avoid uncontrolled proteolysis in cells [74]. Serine proteases of the HtrA family belong to the core set of peptidases and are widely distributed among Gram-negative and Gram-positive bacteria [75]. These proteases have long been linked to inflammation and infectious diseases as the inactivation of htrA genes reduces the virulence properties of diverse bacterial pathogens [76]. These virulence features of HtrA have been linked to lower bacterial fitness, greater susceptibility to stress conditions during infection and/or reduced secretion of virulence factors. HtrA proteases may be exposed to the extracellular milieu as well and elicit bacterial colonization and invasion of host tissues in specific pathogens such as *Bacillus anthracis*, *Borrelia burgdorferi*, *Campylobacter jejuni* and *Helicobacter pylori* [77,78,79,80]. These bacterial pathogens are adept at escaping host defenses and surviving in a very hostile environment. Recently, extracellular HtrAs have been involved in the bacterial invasion process by directly targeting extracellular matrix components, proteoglycans and junctional proteins [81]. HtrA-mediated cleavage of E-cadherin was reported for *C. jejuni*, *H. pylori* and *E. coli* as well [82,83,84,85]. At the molecular level, HtrA proteases target the calcium-binding sites across specific repeats in the extracellular E-cadherin domain [86]. The loss of this protein has been closely linked to intestinal barrier dysfunction, a common feature of IBD. Other microbial serine proteases that have been involved in pathogen–host interactions include VaT-AIEC from AIEC. VaT-AIEC is involved in bacterial adhesion and invasion of host intestinal cells, which further contributes to in vivo pathogenesis [87]. Serine protease autotransporters (SPATE) secreted by enterohemorrhagic *E. coli* (EHEC) are believed to contribute to IBD pathogenicity as well via the proteolytic cleavage of mucin and the degradation of coagulation factor V—the latter effect potentially exacerbates hemorrhagic colitis [88,89].

Proteolysis has been adopted by non-virulent bacteria as well and contributes to gut inflammation. For instance, Subtilisin, a serine protease produced by the non-pathogenic *Bacillus subtilis*, has been shown to activate prothrombin and trigger platelet aggregation and plasma clotting [90] and may contribute to the increased risk of thromboembolic events reported in patients with IBD [91]. 

Besides serine proteases, other effectors that merit consideration comprise gut cysteine proteases. Among the most recognized clans in prokaryotic cysteine proteases, a single family of cysteine exopeptidases, C40, constitutes more than 30% of all cysteine proteases detected so far [92]. These proteases contribute to peptidoglycan turnover and serve as key virulence factors targeting specific components of the host defense system. The role of gingipains and streptopain as key virulence factors of *Porphyromonas gingivalis* and *Streptococcus pyogenes*, for instance, includes the (i) activation of the kinin system [93], (ii) degradation of antibacterial peptides such as human α- and β-defensins [94], (iii) dysregulation of cytokine-signaling pathways [95] and (iv) activation of matrix metalloproteases (MMPs), such as MMP-2 [96]. Although gingipains are mainly linked to periodontal disease, previous studies have highlighted the complex pathogenic interactions between this disease and IBD [97,98]. The administration of *P. gingivalis* altered the composition of the gut microbiota and reduced the expression of the junctional proteins involved in intestinal permeability [99]. IL-6 and TNFα expression was also increased [99].

These proteases offer a promising opportunity for therapeutic intervention in inflammatory and infectious diseases. However, limited data are available regarding the protease/serpin interactions.

## 5. Serpins, Natural Inhibitors to Control the Activity of Serine Proteases

### 5.1. Overview of Serpins

Serine protease inhibitors, also known as serpins, constitute the largest and most widely distributed superfamily of protease inhibitors. It bears over 3000 serpins identified in all living kingdoms, including animals, plants, fungi, protists, archaea and bacteria [100,101]. They generally consist of 350–400 amino acid residues with a molecular weight between 40 and 100 kDa and fold into 7–9 α helices and three β-sheets [102]. Their structure is highly conserved, which is important for their function. Besides their inhibitory roles, serpins serve as hormone transporters [103,104], chaperones [100] as well as antiangiogenic factors [105]. The mechanism of action of serpin-inhibiting proteases comprises a unique conformational change of both molecules and the formation of a suicide complex often referred to as a “mouse trap” [106]. Based on their phylogenetic relationships, serpins can be subdivided into 16 groups named from A to P [102,107]. They often inhibit serine proteases but may also target caspases [108] and papain-like cysteine proteases [109,110]. These inhibitors have been extensively studied in eukaryotes. Indeed, a total of 37 serpins have been identified in humans, 30 of which are functional protease inhibitors [101,111,112]. They are involved in the control of various physiological processes, such as blood coagulation (anti-thrombin), inflammatory responses (anti-trypsin, anti-chymotrypsin) and tissue remodeling [100,113,114]. Unlike eukaryotic serpins, their prokaryotic counterparts are relatively enigmatic. In vitro studies showed that this protein family exhibits inhibitory potential [115]; however, its in vivo targets remain to be characterized. Interestingly, several inhibitory prokaryotic serpins are found in extremophile bacteria, such as serpin from *Pyrobaculum neutrophilum* [116]. These serpins are known to act as inhibitors at elevated temperatures while resisting inappropriate conformational change. 

### 5.2. Non-Gut Microbial Serpins

Prokaryotic serpins have been gaining interest, as indicated by the growing number of serpin-encoding genes since 2013, increasing from 445 to 53,367 [117]. These inhibitors account for 31 and 13% of the fully sequenced genomes of archaea and bacteria, respectively [118,119]. They were first discovered in 2002 when Irving et al. (2002) characterized 12 serpin-like sequences in the genomes of some archaea and extremophile bacteria [120]. The origin of these proteins and their physiological role in prokaryotes remain to be elucidated. We previously analyzed sequences of microbial serpins available in NCBI and demonstrated that these inhibitors are sparsely distributed in different phyla, mainly Actinobacteria, Firmicutes, Bacteroidetes and Proteobacteria. This analysis indicates that serpins belong mostly to the human gut microbiota as well as marine and soil bacteria [121]. Owing to the presence of serpins in commensal and pathogenic prokaryotes that coexist with eukaryotes, it has been proposed that prokaryotic serpins were acquired from eukaryotes through horizontal gene transfer [119,120,122,123]. However, the occurrence of marine and soil bacteria-harboring serpins does not support this statement. Thus, an alternative hypothesis was proposed suggesting that serpins constitute an ancient superfamily that firstly appeared in prokaryotes prior to divergent evolution [120]. This hypothesis was supported by a recent phylogenetic analysis using 6000 non-redundant sequences that encompass serpins from all living kingdoms [124]. It was reported that most microbial serpins belong to two main groups (T and U), where a large proportion exhibits a predicted inhibitory function [124].

Only a few microbial serpins have been subjected to a functional characterization. Thermopin from *Thermobida fusca* was the first to be studied. Although *T. fusca* is a moderate thermophilic bacterium (optimum growth temperature: 55 °C), thermopin was predicted to inhibit proteases [120]. This function was further confirmed by the ability to inhibit chymotrypsin and the formation of a covalent complex, a typical feature of an inhibitory serpin, with the targeted protease [115]. Thermopin is thermostable at 60 °C, a temperature incompatible with the metastable folding of inhibitory serpins (such as α1AT) [115]. Interestingly, structural adaptation to high temperatures allows the thermopin to fold properly and preserve its inhibitory activity at high temperatures [115]. Of note, thermopin represents the only functionally characterized microbial serpin belonging to the group U [124]. Similar observations were reported with *Thermoanaerobacter tengcongensis*, an extremophile bacterium isolated from a hot spring (optimum growth temperature: 75 °C). *T. tengcongensis* encodes an inhibitory serpin, tengpin, belonging to the group T, that inhibits NE and is able to form covalent complex [124,125]. Serpins were also characterized from the soil bacterium *Clostridium thermocellum* known to degrade cellulose. *C. thermocellum* encodes two serpins, namely PinA and PinB. PinA inhibits subtilisin type XXIV, savinase and esperase [126,127]. Since subtilisin-like proteases are present in several *C. thermocellum* genomes and are highly abundant in soil, it was suggested that *C. thermocellum* uses PinA to protect cellulosome from both endogenous and exogenous protease attacks [126,127]. *C. thermocellum* serpin 1270 was shown to inhibit proteases from more than one structural class, a so-called ‘cross-class inhibition’ feature. In addition to inhibiting serine proteases (subtilisin, trypsin, chymotrypsin), this serpin targets papain, which is a cysteine protease [128]. 

Genome analysis of *Gloeobacter violaceus*, isolated from soil and fresh water, demonstrated the presence of a serpin-encoding gene. The expressed serpin, called vioserpin, shares heparin-binding sites with eukaryotic serpins such as kallistatin and thrombin. Vioserpin efficiently inhibits trypsin-like activity and forms a covalent complex [129]. Recently, a new serpin (PI-QT) was identified from the metagenome of a sponge-associated microorganism. PI-QT inhibits both trypsin and alpha-1-antichymotrypsin [130].

Besides the serpins mentioned above, miropin, a protease inhibitor produced by the periodontopathogen *Tannerella forsythia*, was shown to inhibit not only NE, CatG and trypsin but also microbial proteases such as subtilisin, calpain-like peptidase and gingipain K [123,131]. This wide inhibition spectrum is mainly associated with the presence in the reactive center loop of different cleavage sites, outside the usual P1-P1′ site, which allows the formation of a covalent complex together with structural flexibility during complex formation [123,131]. Recent reports indicate that miropin expression levels correlate with gingipain expression, encoded by *P. gingivalis* [132]. Moreover, miropin efficiently inhibits human plasmin, thereby enabling the bacterium to resist plasmin-mediated fibrinolysis and allow bacterial survival in pathological conditions [133]. Hence, miropin was suggested to mediate bacterial virulence and confer protection against both endogenous and exogenous proteases [131,133].

Although these serpins do exhibit an inhibitory effect, they seem most adapted to extreme conditions and a distinct ecological niche, other than the GIT. It is, therefore, important to explore the gut serpinome and delve further into its role in managing or treating IBD.

### 5.3. Gut Serpinome and Inflammatory Bowel Diseases

The human gut microbiota encodes a large number of serpins, hereafter referred to as the gut serpinome, which mainly encompasses inhibitory functions [121,124]. However, only four serpins were functionally characterized till now. The studied microbial serpins, belonging to *Bifidobacterium longum, Eubacterium siraeum* and *Eubacterium saburreum*, appear to play a role in host–bacterium crosstalk. Bifidobacteria are natural inhabitants of the human gut and are known to display immunomodulatory properties. Members of this genus revealed a large repertoire of genes enabling their adaptation and resistance to the hostile GIT environment [134,135,136]. Among them, serpins are described to be involved in this interaction. Indeed, an early study revealed the presence of serpin-encoding homologs in a small number of bifidobacterial species [135]. Nevertheless, sequencing of the genomes of more bifidobacterial species, together with recent advances made by metagenomic analysis, allowed the identification of several additional Bifidobacterial species-encoding serpins [121,137]. Intriguingly, induced transcription of Bifidobacterial serpins in response to different environmental conditions in the GIT has been highlighted [134,135]. This includes the presence of several prokaryotic and eukaryotic proteases [135,138]. SerpinBL, belonging to *B. longum***,** is able to form a stable covalent complex with fecal proteases from mice. Thus, it was proposed that this serpin is released to protect *B. longum* against surrounding proteases. Hence, SerpinBL confers to the bacterium the advantage to evolve and survive in the competitive intestinal environment [135,139]. In line with this observation, SerpinBL has also the capacity to inhibit NE [139]. Since NE is released by activated neutrophils at the site of intestinal inflammation and plays a pivotal role in several digestive pathologies, such as IBD, it was suggested that SerpinBL secreted during inflammation may modulate host–proteolytic activity [139]. Therefore, this inhibitory activity can contribute to the immunomodulatory properties granted to *B. longum* [140] and may impart a relevant role to this strain in preserving gut homeostasis [141]. The SerpinBL was recently reported to attenuate the activation of enteric neurons, as well, in patients with irritable bowel syndrome (IBS, a pathological condition with low-grade gut inflammation). These results take on more importance when considering that fecal supernatants from IBS patients exhibit increased levels of NE, involved in pain induction. Hence, SerpinBL, through NE inhibition, potentially contributes to pain relief in IBS patient [142]. Reduced anxiety-like behavior in mice with inflamed intestines treated with *B. longum* provides more evidence to confirm this hypothesis [143]. Moreover, it was demonstrated that SerpinBL attenuates gliadin-induced inflammation and impacts intestinal microbial composition in a mouse model of gluten sensitivity [144]. These results stressed the role of *B. longum* in maintaining human gut homeostasis through several mechanisms, including serpin expression. 

*E. saburreum*, another commensal member of the GIT [145], displays saburopin. This serpin preferentially inhibits mammalian pancreatic elastase, prevalent in the GIT, where *E. saburreum* might be encountered [146]. Similar to *E. saburreum*, *E. siraeum*, which naturally colonizes the human intestine [147], was shown to express two distinct serpins named siropin 1 and siropin 2. Siropins efficiently inhibit NE and PR3, two elastase-like proteases abundantly expressed under intestinal inflammation and widely involved in the tissue damage associated with IBD. To the best of our knowledge, siropins are the first microbial serpins to inhibit human PR3. These results suggest an intriguing possibility that *E. siraeum* produces siropins to modulate host-derived proteolytic activity, thereby to resist damage caused by excessive host proteases. In line with this hypothesis, both siropins strongly inhibit fecal proteases harvested from mice with DSS-induced colitis, mainly fecal elastase-like activities. These data highlight the siropins’ potential to inhibit proteases associated with gut inflammation [11]. However, additional analyses are still needed to decipher the impact of *E. siraeum* and its secreted serpins in the gut physiology. Based on the role of the studied serpins in dampening protease-mediating inflammation, it can be assumed that the gut serpinome is potentially involved in the host–gut microbiota interaction, thereby modulating the inflammatory response and the underlying proteolytic pathways as well. Thus, there is growing interest in the gut serpinome as a novel therapeutic alternative against intestinal inflammation. Recent studies have shown that intestinal parasite-derived serpins may contribute to alleviate TNBS-induced colitis in mice [148,149]. *Trichinella spiralis* was found to encode two antiproteases that target digestive serine proteases such as trypsin, chymotrypsin and elastase, as well as cysteine proteases including cathepsin and papain [148,149].

This ability to inhibit two distinct families of proteases highlights the importance of intestinal parasite-derived serpins as members of the gut serpinome. Future studies addressing their contribution to IBD might help to better elucidate the relevance of the gut serpinome in digestive inflammation.

A summary of the different serpin groups and their biological functions is provided in Table 1. 

## 6. Conclusions

The present review highlights the limits of current therapies of IBD and the need for innovative treatments. Apart from the gut microbiota dysbiosis and disbalanced pro/anti-inflammatory cytokines, the proteolytic imbalance appears to exert pivotal functions in the pathogenesis and maintenance of IBD. Here, we focused on the role of the gut serpinome in the microbiota–host dialogue and its impact in maintaining proteolytic homeostasis has been emphasized. Until recently, only four serpins from the gut serpinome had been characterized and their therapeutic potential is being actively investigated. Interestingly, the studied serpins show a large spectrum and high efficiency to inhibit proteases involved in IBD pathophysiology. Such observations stress the enticing prospect of serpins from the gut microbiota in the field of intestinal inflammation. An innovative axis would consist of modulating the microbiota to promote the gut serpinome and thus fight against the deleterious effects of proteases. However, there is quite a dynamic investment in microbial serpins and much effort is still needed to decipher the gut serpinome. Therefore, there is still some way to go before the implementation of this axis because, as underlined in this review, the physiological roles of proteases are vast and any inhibition will have to be targeted. Above all, this review integrates the microbiota into its host, considering the whole as a unique supraorganism (holobiont) with multiple interactions.

## Figures and Tables

**Table 1:** Serpin groups and their main functions.

Clade	Serpin	Biological Functions	References
A	Serpin A1, A3, A4, A5, A10, A12	Serine protease inhibition	[150,151,152,153,154,155]
	Serpin A6, A7 and A8	Hormone transport	[156,157]
B	Serpin B1, B2, B3, B4, B8, B10	Serine and cysteine protease inhibition	[109,158,159,160,161,162]
C	Serpin C1	Inhibition of thrombin, factor Xa and factor IXa	[163,164,165]
D	Serpin D1	Inhibition of thrombin	[166]
E	Serpin E1 and E2	Serine protease inhibition	[167,168]
F	Serpin F2	Inhibition of plasmin	[169]
G	Serpin G1	Inhibition of C1 proteinase and plasma kallikrein	[170,171]
H	Serpin H1	Chaperone	[172]
I	Serpin I1	Inhibition of plasmin, uPA and tPA	[173]
U	Thermopin	Inhibition of chymotrypsin	[115]
T	Siropins, Tengpin, Miropin, SerpinBL	Inhibition of eukaryotic proteases	[11,125,131,139]

## Data Availability

This study do not report any data.
